# Perceptions of self-rated health among stroke survivors: a qualitative study in the United Kingdom

**DOI:** 10.1186/s12877-018-0765-8

**Published:** 2018-04-02

**Authors:** N. Mavaddat, E. Sadler, L. Lim, K. Williams, E. Warburton, A. L. Kinmonth, J. Mant, J. Burt, C. McKevitt

**Affiliations:** 1 Division of General Practice, School of Medicine, University of Western Australia, 35 Stirling Highway, Crawley, Perth, WA 6009 Australia; 2 Department of Public Health and Primary Care, University of Cambridge, Strangeways Laboratory, 2 Worts Causeway, Cambridge, CB1 8RN UK; 3 Health Service & Population Research Department, King’s Improvement Science and Centre for Implementation Science, King’s College London, De Crespigny Park, Denmark Hill, London, SE5 8AF UK; 4 Department of Clinical Neurosciences, University of Cambridge, Neurology Unit, R3, Box 83, Cambridge Biomedical Campus, Cambridge, CB2 0QQ UK; 5 Department of Public Health and Primary Care, University of Cambridge, Forvie Site, Robinson Way, Cambridge, UK; 6 School of Population Health Sciences, King’s College London, Addison House, London, SE1 1UL UK

**Keywords:** Stroke, Self-rated health, Disability, Rehabilitation, Qualitative research

## Abstract

**Background:**

Self-rated health predicts health outcomes independently of levels of disability or mood. Little is known about what influences the subjective health experience of stroke survivors. Our aim was to investigate stroke survivors’ perceptions of self-rated health, with the intention of informing the design of interventions that may improve their subjective health experience.

**Methods:**

We conducted semi-structured interviews with a purposive sample of 28 stroke survivors recruited from a stroke unit and follow-up outpatient clinic, 4–6 months after stroke, to explore what factors are perceived to be part of self-rated health in the early stages of recovery. Qualitative data were analysed using a thematic analysis approach to identify underlying themes.

**Results:**

Participants’ accounts show that stroke survivors’ perceptions of self-rated health are multifactorial, comprising physical, psychological and social components. Views on future recovery after stroke play a role in present health experience and are shaped by psychosocial resources that are influenced by past experiences of ill-health, dispositional outlook such as degree of optimism, a sense of control and views on ageing.

**Conclusions:**

Severity of physical limitations alone does not influence perceptions of self-rated health among stroke survivors. Self-rated health in stroke survivors is a multidimensional construct shaped by changes in health status occurring after the stroke, individual characteristics and social context. Understanding the factors stroke survivors themselves associate with better health will inform the development of effective approaches to improve rehabilitation and recovery after stroke.

## Background

Patient-reported outcome measures such as self-rated health, are an important component in the assessment of health status [1, 2]. Self-rated health (SRH) predicts a range of future health outcomes across populations and especially in older people, including functional decline, health care use, and institutionalisation [3–9]. Following a stroke, comparative measures of SRH predict functional outcome, stroke recurrence and mortality after adjusting for disability [10]. Patient-centred measures therefore capture information additional to that obtained from objective predictors of outcome, such as type and severity of stroke [11–13], and facilitate a more holistic patient-centred assessment of post-stroke recovery.

The presence of a stroke is generally associated with a poorer SRH compared to that seen in other older people; and a reduction in SRH from pre-stroke status [13, 14]. For some stroke survivors, this effect is temporary, but for others it persists. However, the longer-term impact of stroke on SRH may be less directly related to the severity of the stroke than may be anticipated [4, 10, 15–18] - a kind of ‘disability paradox’. The concept of a ‘disability paradox’ was coined by Albrecht and Devlieger in 1989 based on their observation that those with physical disability often report a surprisingly good quality of life [19]. However in stroke survivors, the concept of a disability paradox has not been adequately explored. The presence of such a paradox would suggest that factors besides physical disability and severity of stroke influence the subjective health experience in the longer-term.

In a previous cross-sectional quantitative study we conducted of over 700 stroke survivors from the Medical Research Council Cognitive Function and Ageing Study (MRC-CFAS), psychosocial factors including depression and not “getting out and about” were found to be important determinants of SRH in patients who have had a stroke [14]. Understanding what factors shape perceptions of SRH among stroke survivors, particularly in the context of disability, and which factors might be potentially modifiable in the subjective health experiences, will aid the development of better models of post-stroke care, and more effective rehabilitation. Stroke survivors have often reported post-stroke discharge services as being inadequate [20], so a focus on interventions that address stroke survivors’ subjective experiences of the impact of stroke are needed. Improving stroke survivors’ wellbeing and subjective experiences of their own health is not only an important goal in itself, but may potentially impact more objective stroke outcomes.

In this study we use a qualitative approach to gain depth and insight into stroke survivors own perceptions and experiences of their health and investigate what modifiable and non-modifiable factors may influence the subjective health experience in the earlier stages of recovery following a stroke.

## Methods

### Recruitment strategy

Potential participants were identified from a rehabilitation stroke unit at Cambridge University NHS Foundation Trust Hospital and by a stroke consultant or a specialist stroke nurse at a follow-up outpatient clinic. We used a purposive sampling approach, and aimed to recruit participants from a range of ages and levels of disability. We included individuals with a first confirmed stroke, who had the ability to take part in an interview and provide their informed consent. We excluded stroke survivors with severe clinical aphasia and cognitive impairments (clinically assessed as a Mini Mental State Examination (MMSE) score of less than 20) [21], and those who did not speak English.

Age, gender and postcode were obtained for all participants by the study team. The Index of Multiple Deprivation (IMD) [22] was derived from participant postcodes as a proxy for socioeconomic status. Physical disability was assessed using the Modified Barthel Index of Activities of Daily Living (BI) [23], taken either at hospital discharge or on attendance at a 6 week follow-up outpatient clinic. A score of 18 or above (equivalent to greater than 91 out of 100 on the original BI) was considered ‘minimal’ disability (‘none’ or ‘slight’ dependence on the BI), and a score of 17 or below as ‘significant’ disability (‘moderate’, ‘severe’ or ‘total’ dependence on BI) [24]. Participants were asked the single Self-Rated Health (SRH) question: “How would you rate your general health?” Response options were given on a 5 point Likert scale: ‘very poor’, ‘poor’, ‘fair’, ‘good’ and ‘excellent’. They were also asked to complete the Hospital Anxiety and Depression Scale (HADS) [25]. Number of co-morbidities defined as co-existent long-term conditions other than the stroke were also recorded by reviewing clinical notes.

### Data collection

Ethical approval for the study was obtained from the National Health Service (NHS East of England) – Norfolk REC (ref 11/EE/0108). We obtained written informed consent from all participants prior to taking part in interviews. Semi-structured interviews were conducted with stroke survivors to investigate what factors they perceived contributed to their subjective health. LL, ES and NM interviewed participants over a 10-month period between September 2011 and June 2012. Interviews took place in their own homes, between 4 and 6 months following their stroke. Informal carers, often spouses, were present in one third of the interviews, based on participants’ preference. Interviews lasted between 45 and 80 min.

The interview topic guide was developed based on review of existing qualitative studies exploring perceptions of wellbeing and quality of life among stroke survivors and analysis of two prior focus groups with stroke survivors from a local Stroke Association patient group (unpublished). During the interviews, stroke survivors were asked questions about their health, including their perceptions of recovery and health after stroke; the impact of the stroke on daily life and social relationships; the quality and nature of relationships with health care professionals; and their views about future health and recovery.

### Data analysis

All interviews were audio-recorded, transcribed verbatim and then stored, managed and coded in NVivo (Version 9.0) Computer Aided Qualitative Data Analysis Software. Transcripts were first read and re-read by NM and LL and coded for themes using a thematic analysis approach to identify similarities and differences in themes between participants [26]. Coding categorisations were developed both from those identified a priori through topics covered within the interview guide (e.g. family support and access to health care services as part of participants’ self-perceptions of health), and from those emerging inductively through the course of the interviews. Themes were further analysed with two authors (CM and ES) contributing to the analysis by reading a selection of transcripts and offering suggestions on the developing coding framework. Data collection and analysis proceeded in an iterative manner until no new themes were identified from the interviews [27].

## Results

Twenty-eight stroke survivors were interviewed (age range 47–86 years), comprising 19 men and 9 women. Participant characteristics including SRH responses, socio-demographic characteristics and number of co-morbidities are shown in Table 1. Six participants rated their health as excellent, 15 as good and 6 as fair, with only one stroke survivor rating their health as very poor. Eight participants had ‘significant’ and 21 had ‘minimal’ levels of disability on the BI. Most stroke survivors had at least one other co-morbidity, including hypertension, diabetes, musculoskeletal conditions (arthritis and fractures), chronic gastrointestinal or respiratory problems and cancer. Half of participants were in the least socially deprived IMD (top quintile) category. A ‘disability paradox’ was evident in eight stroke survivors who reported ‘excellent’ or ‘good’ SRH despite experiencing ‘significant’ disabilities after stroke. In contrast, 6 out of 15 of those with ‘minimal’ disabilities reported ‘fair’, and one ‘very poor’ SRH.

**Table 1:** Socio-demographic characteristics and psychological status of 28 study participants with stroke

Study ID	Gender	Age Range	Disability^a^	Self-rated Health (SRH)	Number of Comorbidities	Index of Multiple Deprivation (IMD)^b^	Depression Score (HADS-D)^c^	Anxiety Score (HADS-A)^d^
Mr A	M	≥85	Significant	Good	Two	One	None	None
Mrs B	F	65–74	Minimal	Fair	Four	Two	None	None
Mr C	M	65–74	Minimal	Good	Three	One	None	None
Mr D	M	55–64	Significant	Excellent	Three	One	Moderate	Significant
Mrs E	F	45–54	Minimal	Fair	Three	Three	Moderate	Significant
Mr F	M	≥85	Significant	Good	Three	One	None	None
Mr G	M	65–74	Minimal	Good	Two	One	None	None
Mr H	M	75–84	Significant	Good	Two	One	None	None
Mr I	M	75–84	Minimal	Fair	Two	Four	None	Moderate
Mr J	M	55–64	Minimal	Good	One	Two	None	None
Mrs K	F	45–54	Significant	Good	Two	Three	None	Significant
Mrs L	F	75–84	Minimal	Good	Three	One	None	None
Mr M	M	65–74	Minimal	Excellent	Two	Two	None	None
Mr N	M	55–64	Minimal	Good	Three	One	None	None
Mr O	M	65–74	Minimal	Good	Two	Two	None	None
Mr P	M	55–64	Significant	Excellent	Two	Two	None	Moderate
Mrs Q	F	75–84	Minimal	Good	Two	Two	None	None
Mr R	M	45–54	Minimal	Fair	One	One	None	Moderate
Mr S	M	75–84	Minimal	Excellent	Two	One	None	None
Mr T	M	65–74	Minimal	Excellent	Three	One	None	None
Mrs U	F	55–64	Significant	Good	Four	Four	None	None
Mrs V	F	45–54	Minimal	Good	Four	Two	None	Moderate
Mr W	M	75–84	Significant	Good	Three	One	Moderate	Moderate
Mrs X	F	75–84	Minimal	Good	Two	Two	None	None
Mr Y	M	65–74	Minimal	Fair	One	Three	None	None
Mr Z	M	65–74	Minimal	Fair	Two	One	None	Moderate
Mr AA	M	65–74	Minimal	Excellent	One	Four	None	None
Mrs BB	F	55–64	Minimal	Very poor	Two	One	Significant	Significant

^a^Minimal disability BI > = 18, Significant disability BI = < 17

^b^IMD 1 = top 5 = lowest

^c^HADS-D Hospital and Anxiety Depression Scale - Depression

^d^HADS-A Hospital and Anxiety Depression Scale - Anxiety

**Fig. 1:**
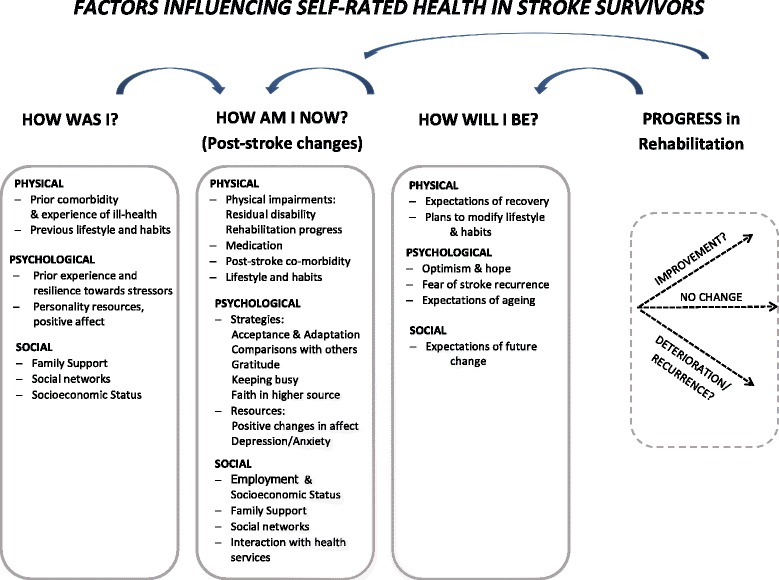
Factors influencing self-rated health in stroke survivors

Our analysis identified a number of themes describing stroke survivors’ perceptions of their own health following the stroke (see fig. 1). These included reflections on their present health status and circumstances, but also past experiences and future outlook. In thinking about their health, stroke survivors broadly considered: “How am I now?” “How was I before?” and “How will I be in the future”? In the supporting quotations below, pseudonyms are provided to protect the anonymity of participants. In supporting dialogue extracts ‘I’ indicates ‘interviewer’ and ‘P’ indicates ‘participant’.

### How am I now?

#### Physical influences

For participants with physical disabilities following their stroke, impairments such as limb paralysis and aphasia were key influences on reports of SRH. Such limitations impacted on the ability to carry out activities of daily living, work and leisure activities. The desire to return as much as possible to ‘normality’ i.e. pre-stroke functioning, and progress in rehabilitation, were key concerns for these stroke survivors. Among those with no or minimal physical disabilities, self-appraisals of current health status were often closely tied to their perceptions of having made good progress with their recovery. Participants with physical disability commonly identified practical solutions for minimising the impact of residual disabilities on their experiences of health. These included strategies such as performing daily tasks in different ways, for example moving regularly needed objects to lower shelves in the home among those with hemiplegia, or in those with swallowing problems taking smaller portions of food and drink. For instance, Mr A said:

My brain rather is learning how to cope with these restrictions and finding a way round them. So doing the things that I want to do in a slightly different way if you like …… If I use a spoon normally with this hand and you go to put a spoonful of liquid in your mouth, I can’t do that because by the time I get the spoon up near my mouth half the liquid has run out because I can’t control it. If I start off with the spoon half full, yes I can do it. They’re the sort of things that you adapt to. (Mr A, ≥85 years)

Perceptions of self-rated health among stroke survivors were also negatively shaped by experiences of post-stroke pain in some participants, and in a greater number of participants by ongoing stroke-related fatigue. For example, Mrs B commented:

I: When you said your health was average, what kind of things went through your mind?

P: I get cross with myself because I think, oh why can’t I do that … but then I know that if I do too much then my knee will be so painful, I’ll be up all night, so I tend to sit…Well I’m not as able bodied as I was, I get tired quicker. (Mrs B, 65-74 years)

Stroke survivors commonly reported co-morbidities, some of which had developed after their stroke, such as a fall-related fracture or frozen shoulder. Such disabilities had interrupted or significantly slowed down their rehabilitation and progress with recovery, and impacted on current perceptions of self-rated health. The need to take daily medication following a stroke, including medication to reduce blood pressure and cholesterol levels, and the need for multiple attendances at medical and other appointments also negatively affected perceived current health status. For example, Mr C said:

I: When you think about your health do you think having had the stroke changes how you feel in yourself about your health?

P: It has to a bit because I find I’m getting endless hospital appointments. There’s the stroke and then I had some urology issues *…* (Mr C, 65-74 years)

Other physical influences shaping current perceptions of SRH among stroke survivors included diet and level of exercise. A quarter of participants reported making changes to their diet and exercise following the stroke, and reducing alcohol and smoking, as strategies to feel healthier and minimise risk of stroke recurrence. A few also felt that they had an improved understanding of their physical health since their stroke, including a greater awareness of their body, being less willing to stress themselves physically, and to cherish their health. One man reported:

I: Has the stroke made you think about your health in a different way?

P: “I think so, yes, yes, ... I am much more free and easy with my health.” (Mr D, 65-64 years)

#### Psychological influences

Mental health status, cognitive problems in relation to memory and concentration, and changes in mood and affect (including irritability, impatience, worry, anxiety and feeling low) all impacted negatively on SRH. Reporting of anxiety or depression was common in both those with minimal and significant physical disabilities and reported by at least one third of participants. For example, one middle-aged woman Mrs E, said:

Well …it does affect your attitude and your moods .…Mentally, I have had some down times, it was a little shocking for me to have this, because I’ve always been a very confident person. (Mrs E, 45-54 years)

Low mood was perceived to be mostly related to loss of independence, changes in occupational and social status, slow progress in rehabilitation and loss of confidence. For example, this included Mr C, who despite making a good physical recovery, lacked confidence in being able to carry out some of his usual activities, including taking part in sports and travelling on his own:

I’m superficially in good health. Superficially. There's a few things underneath which are, you know, undermining my confidence. (Mr C, 65-74 years)

In contrast, approximately one third of participants reported psychological changes that positively impacted their feelings of wellbeing and subjective health, such as becoming more patient, content, tolerant, thoughtful, appreciative, and valuing the smaller things in life such as time spent with family, friends and neighbours. Such changes were mostly the result of a modification of psychological outlook in response to what was perceived as a serious threat to their health after experiencing a stroke.

Almost all stroke survivors, in particular those with significant physical disabilities, described a range of psychological strategies to cope with the psychological impact of their physical limitations following the stroke, and positively appraise their own health. A commonly used strategy in over half of the participants, including those with significant disability, was making comparisons with others, both those perceived to be less fortunate, and those who had made a good recovery from the stroke, with the latter seen as a source of inspiration and motivation for their own recovery. Some participants also compared themselves with others of their own age, feeling themselves to be in relatively good health despite their stroke. Many reported being grateful for being alive, for their remaining physical abilities and progress in rehabilitation:

I: So you said your health’s not bad?

P: I don’t think so.

I: What makes you say that?

P: Well see I mean it depends what you add up. I mean if I sit there thinking of what I’ve had and what I’ve got, I go into decline, but I think well, you know, I don’t think it is bad, I think I’m given a lot of grace to pull myself together and do things, and as long as I can do that then my health’s alright. (Mr F, ≥85 years)

Other coping strategies used by stroke survivors to appraise their health in a positive light, included having life goals and motivations for their recovery and keeping busy with activities, such as returning to usual hobbies and activities. Determination, hard work and perseverance with goals, having a sense of control, taking personal responsibility, striving for independence and a lack of reliance on others were all psychological strategies adopted by these participants to appraise their health and perceptions of future progress with recovery and control over their health positively. For example, one man and one woman said:

I: And what’s got you here to this point?

P: Yeah. If at first you don’t succeed, yeah. Determination…(Mrs BB, 55-64 years)

Don’t let people mollycoddle you, just get up and go and do what you have to do. If you want a cup of tea, go and make a cup of tea, that sort of thing. (Mr G, 65-74 years)

For some stroke survivors with significant disabilities, psychological acceptance of current physical limitations following the stroke, but also other co-morbidities prior to the stroke and the ageing process, were important factors mediating perceptions of SRH. A couple of participants who positively coped with physical disabilities also spoke of having a strong faith in a God or a higher power to whom they entrusted their health and recovery. For example, Mr. F said:

I think faith helps an enormous amount. Yeah… I do believe that we have a Father who looks after us… I don’t know how people can live without faith to be honest, so that is an enormous comfort to me. … I sort of think now give a little time and it will come back and he’ll make the hand work again so (laughter) … Yeah I don’t expect miracles but I do know I get miracles… (Mr F, ≥85 years)

#### Social influences

The majority of stroke survivors interviewed reported a number of social influences shaping perceptions of SRH in both positive and negative ways. These included their immediate home environment, work, family, social circumstances and their ability to get out and about.

I enjoy my music, I enjoy reading, of course I like being with my wife, I have my garden. What’s going on around my environment really keeps me healthy. (Mr. H, 75-84 years)

For half of stroke survivors, changes in circumstances, such as being unable to work, the loss of roles inside and outside the home, and inability to carry out hobbies and leisure activities, impacted on reports of their overall health and particularly, mental health:

It’s just getting used to not doing anything. You know, I’ve worked all my life and then suddenly you’re not doing anything, you sort of find… I had an allotment. I was going to carry on with that, but I found it’s too much to get down there and trying to do things, you know, and then you get tired quite quickly too, you know … It’s getting around and not having a car anymore, you know. I’ve got to go to town, I’ve got to walk there and [that] sort of thing. I had to go to the doctor the other day and I had to walk there and it rained, so I got soaking wet. You know, that sort of thing, but, ah, it’s part of life, you know, so it must carry on. (Mr G, 65-74 years)

The quality of social relationships was an important factor shaping perceptions of SRH among stroke survivors. The presence of a supportive spouse and family members were particularly important: having regular contact with family members, including children and grandchildren, was one of the most important sources of mental wellbeing and enhanced experiences of health identified by stroke survivors. Two participants reported positive changes in family relationships following the stroke, for example, with siblings or children becoming emotionally closer, while none reported a worsening of relationships. The majority of stroke survivors identified family members as key sources of social support and encouragement when undertaking activities of daily living, exercises as part of their ongoing rehabilitation, following advice with regards to making lifestyle changes, and getting ‘out and about’. For example, one man said:

If you’ve got a good family they’re the best medicine in the world to make you sit up. I mean my wife or my son won’t let me mope. (Mr I, 75-84 years)

A minority of stroke survivors reported a lack of close family support after the stroke, which had a negative impact on their perceived mental health. Specifically, two women spoke about a lack of emotional and practical support from their spouse. A number of other stroke survivors also described family members as being over-protective and trying to limit them from carrying out daily tasks in case they got tired, which similarly had a negative impact on their current experiences of health.

The majority of participants reported changes in the type and quality of their social relationships after stroke, which they spoke about in the context of their health. For some, friends and neighbours became more supportive and helpful, for example in terms of visiting more often and helping with tasks, although others reported friends and neighbours subsequently avoiding them. Those with minimal physical disability spoke of challenges with ‘invisible’ disabilities, such as being unable to concentrate and tiring easily in their interaction with others.

Several stroke survivors attributed their good health to the quality of health care they had received, including timely interventions and positive interactions with healthcare professionals who were perceived to be caring and supportive. They often praised the good work of hospital and community staff, but felt that poor support given by primary care when discharged from hospital had had a negative impact on their health and recovery. Rehabilitation therapists had played a greater part in discussions about health among those with more significant disabilities. However, there was little difference reported in perceptions of satisfaction with health services among those with differing degrees of disability. One man, Mr H, with a significant disability after his stroke typically said:

My health… in general... pretty good, well looked after, I put it down to that, and the attention that’s been given to me, principally by the hospital (Mr H, 75-84 years)

### How was I before?

Participants’ accounts of their current SRH often included reference to their lives and previous health before the stroke, across the same physical, psychological, and social domains.

#### Physical influences

The majority of stroke survivors, especially those of older age, reported living with long-term conditions prior to their stroke, in particular diabetes, hypertension, arthritis or cancer. Stroke onset was largely perceived as adding a further significant burden to their current health, both physically and emotionally. For example, one female participant, Mrs. E, said:

Average [health], not fantastic ‘also’ because of the diabetes and since I’ve had cancer I haven’t had the stamina that I had before, so I would say it [current health] was just average really. (Mrs B,65-74 years)

Similarly, another woman commented:

P: I have insulin-dependent diabetes, I have kidney disease, I have heart disease and now I’ve had a stroke, I’ve had retinopathy, so if we’re ticking off the boxes for complications from diabetes, I think I pretty much have them all (laughs). And so, even if I were in the most excellent of health with my diabetes, these other complications that I have would still put me only in mediocre health, I don’t think I’ll ever have excellent health again (laughs).

I: Right and did the stroke change your perception of your overall health?

P: Oh no, it just was one more box to tick off in the complications. (Mrs E, 45-54 years)

Previous lifestyle habits were considered among a third of stroke survivors, including both men and women, as having an impact on perceptions of SRH, either by contributing to the development of stroke, or conversely, by positively impacting upon current health post-stroke. For example, one man said:

I try and eat well and do plenty of exercise, yeah I think I’m as healthy as any, if not healthier than any late 50 year old to be honest, yeah, yeah … But yeah as the consultant said, he said because I was, I’m pretty fit that’s helped me over the stroke really, you know. (Mr J, 55-64 years)

#### Psychological influences

Stroke survivors often identified aspects of their personality and life history (particularly difficulties in childhood) as influencing their current outlook, resilience and beliefs about their health and in their ability to overcome the challenges of rehabilitation. Two participants, one man and one a woman, felt that their experiences of having coped with prior illness or in looking after other family members or friends had shaped their ability to cope positively with the impact of their stroke currently:

I really wasn’t too upset about it because I’d had such a bad time with my husband two years previously who had had a stroke. (Mrs K, ≥85 years)

Several participants when describing their health also reported that that they were ‘upbeat’, naturally happy and content within themselves, and this positively shaped perceptions of their health following the stroke. For example, one man, Mr H, reported in the context of being happy with other aspects of his life including his wife’s support, and despite experiencing significant stroke-related disabilities and other comorbidities including cancer, that this did not get him down:

Your health, you can forget it, you know, you can, and as I say, from the stroke and everything in general, I’m exceptionally happy. (Mr H, 75-84 years)

Almost all stroke survivors believed that a positive disposition and optimism were important to their health and recovery from the stroke. Other positive character dispositions including possessing a self-reliant and independent personality were reported by almost a third of participants, which shaped their ability to cope with their rehabilitation after the stroke. A number of these participants also spoke of having been resilient in the face of adversity throughout their lives. For example, one man and one woman considered that their resilient characters shaped their current positive outlook on their health following their stroke:

My parents, particularly my mother, you know, you can either be defeated or you can get over this. She was never very sympathetic if anything went wrong that was minor, you know, “Get over it, you know, you’ve got to learn to handle it, it’s up to you, you can get wherever,” but she was always there as a tremendous support but I do think it was the way I was brought up. (Mrs L, 75-84 years)

I: What do you think’s made you be a positive person?

P: Because I had to fight for when I was a little’un, I think during the war I was a bit of a bugger, I, when the doodlebugs used to fall whenever they hit we used to run around and find what we could, you know, furniture, bits of shrapnel and then we were brought up in an orphanage, I had to be positive, you know. (Mr I, 75-84 years)

In contrast, two other participants, both of whom were men, reported that they had always had negative psychological traits such as being impatient, which they perceived made their rehabilitation and return to health after their stroke more challenging.

#### Social influences

Participants reported that a number of pre-existing social factors had positively impacted on their subjective health experiences and wellbeing following the stroke. These included prior financial stability, enjoying work, a positive home environment, supportive family and friends and the ability to continue with social activities and to travel, which were all sustained after the stroke. For example, one man said:

I do a lot of walking. I go and visit different towns just to get out and do something really.......I went on holiday in February, I went to the Gambia for 12 days. I’m going off to Singapore and to Borneo in September for 17 days. .......Well, I don’t lack anything that I feel that I need. I’m not short of a few shillings, I have lots of people around me that are great friends, I have a good life, I can do whatever I want to do. (Mr M, 65-74 years)

### How will I be in the future?

#### Physical and psychological influences

Participants looked to the future when reflecting on their current health status after the stroke. Expectations of recovery from stroke-related disabilities and perceived rate of progress of improvement in physical function were intimately connected with perceptions of mental health and wellbeing. A minority of stroke survivors, however, also feared the process of getting older, and worried that this would negatively impact on their ability to return to independent physical functioning. However, the majority of stroke survivors were willing to accept the impact of the ageing process on their health and preferred to take ‘life as it comes’. This included more than half of participants with significant disabilities, including those with significant hemiplegia who reported that being optimistic was central to their health and future recovery. Five participants, reflecting four men and one women, who did not feel that they were inherently optimistic, nevertheless tried to be positive and hopeful about their future recovery and improvements in health status. For example, one man, Mr N said:

I just feel so totally and utterly frustrated and I kept doing me own exercises ... but my policy is it’s happened, I’ve got to get on with life as best I can … You’ve got to have a positive outlook I think, that’s all I can say really, yeah. (Mr N, 55-64 years)

In contrast, nearly one third of participants described ongoing anxiety and concern about the possibilities of poor physical recovery and potential stroke recurrence, having a negative impact on their perceptions of SRH and wellbeing. For example, one woman Mrs B, commented:

Well supposing I had another one [stroke], although they said it was very unlikely but supposing I had another one and I was on my own ... I’m aware if I don’t feel 100% that I think, oh dear, am I going to get another one … (Mrs B, 65-74 years)

#### Social influences

Many stroke survivors voiced that they may have been influenced in their views of their own health by family, friends and healthcare professionals’ projections regarding the potential for their future recovery. One man commented:

When they watched me struggling to get on the bed, I suppose, ‘Ooh, you are doing well. Good. Jolly good. You’re coming along beautifully with your physio and everything. You’re making progress. You’re so positive and strong’ and really wonderful ... my friends tell me I’m an old bugger who’ll live forever.… (Mr M, 65-74 years)

A few stroke survivors also looked to the future with hope of improvements in their financial position and social participation with regards to returning to work, and other activities such as driving, hobbies and interests. Feeling healthier in the future was linked by some to a potential positive change in social circumstances.

I: Is there anything that you think would make you feel healthier?

P: Well, my car. (laughs) … (Mr O, 55-64 years)

P: Yeah, winning the lottery. (laughs) (Mr P, 55-64 years)

## Discussion

This is the first qualitative study to explore in-depth the self-rated health experiences of stroke survivors. We found perceptions of SRH among stroke survivors in our study to be multidimensional, and both positively and negatively shaped by physical (e.g. comorbidities, lifestyle factors), psychological (e.g. depression, presence or absence of psychological coping strategies), and social dimensions (e.g. quality of family support, interactions with healthcare professionals and access to services). Our findings, whilst seemingly less evident than among individuals with more stable and long-standing disabilities, such as in spinal cord injury [28], point to a ‘disability paradox’ observed in the perceptions and experiences of some stroke survivors interviewed, who perceived their SRH positively despite significant disability. Conversely, some stroke survivors with minimal disabilities had negative perceptions concerning their health. The impact of physical disability on subjective appraisals of health differed widely among stroke survivors in this study, and was apparently moderated by a range of individual factors and social resources. On the one hand, whatever the immediate impact of sudden physical disability, such resources may counter in the long-term, some of the negative experiences of having had a stroke. On the other hand, among those who did not have such positive resources, personal or social circumstances, even minor disability brought on by a stroke may have negative implications for perceptions of health.

There is significant overlap between our findings and the existing literature on self-rated health in the wider population. Indeed, physical functioning and social influences are known to modify subjective health perceptions in the general population and among those with long-term conditions [15, 29–40]. The presence of multiple morbidities and lifestyle factors are known to impact perceptions of SRH [39, 41]. Psychological disposition, mental health issues such as anxiety and depression, and personality characteristics are also known to influence SRH [32, 42]. Studies particularly suggest that a sense of control over one’s own health may be important in appraising subjective health experiences in a range of patient groups [32, 42]. Perceptions of SRH among stroke survivors in our study, however, extended well beyond a reflection of present physical and mental health and social circumstances. In stroke survivors, past experiences including prior experiences of health, as well as future outlook and assessment of the potential for recovery appeared important elements of the health experience. Assessments of past and predictions of future health are known to influence SRH perceptions in the population [43, 44], but have so far have not been well studied in the stroke population. Understanding factors shaping resilience, coping and a positive outlook on potential stroke recovery are important [45], since they may both positively predict and influence stroke outcomes. Idler suggests that the relationship between SRH and its ability to predict objective health outcomes exists because individuals have “access to information about their current and future health status, survival probabilities, or changes in future risk behaviours that is not obtainable by other means” [44]. Future health expectations, especially among older people, have been found to predict health outcomes at times even more accurately than present health status [44, 46]. The extent to which attitude towards recovery, coupled with the need for hard work and determination were emphasised in this study by participants in relation to their rehabilitation, suggests that beliefs about the potential for physical recovery and the ability to modify this recovery form an important element of the overall subjective health experience of stroke survivors. Thus self-appraisals of health among stroke survivors involve expectations of the ‘time-line’ of physical recovery, and are influenced by perceptions of the ‘controllability’ of progress with rehabilitation through stroke survivors own efforts [47, 48].

Following a stroke, levels of physical impairment in the shorter term are subject to change, have an uncertain trajectory with varied individual responses to rehabilitation, with prediction of rate of recovery and outcome often difficult [49]. The lack of certainty about expected recovery may lead to disappointment in the stroke survivor. Later acceptance and adaptation may modify expectations of recovery and lead to a process of on-going re-assessment and re-adaptation to health status [40, 50, 51]. Nevertheless with the passage of time, more stable underlying factors that moderate SRH in stroke survivors, including personality and outlook, as well as potentially modifiable social factors, may become increasingly important components shaping subjective health experiences and perceptions of wellbeing among stroke survivors.

While the relationship between physical functioning, disability and self-perceptions of health among stroke survivors is complex and strongly mediated by context and individual factors [52], their impact on disability may be diminished and experiences of health enhanced by assessing self-perceptions in individual patients and tailoring rehabilitation accordingly. A simple question of ‘how would you rate your health?’ followed by a range of options ‘(for example, poor, fair, good or excellent), can give a wealth of information to the healthcare professional, and aid in the targeting of interventions. Interventions that potentially enhance subjective health experiences after stroke, such as those that modify the social circumstances of stroke survivors, and those that focus on enhancing psychological outlook and resilience, enabling stroke survivors to cope with the stressors of rehabilitation and take control of their recovery, are likely to be important to improving the overall health status of stroke survivors. These could be offered to those especially with poorer perceived subjective health following a stroke. Further, knowledge of what drives poor but also ‘excellent’ subjective health may aid development of better models of post-stroke rehabilitation and future research.

### Limitations

Stroke survivors with severe stroke-related disabilities and poor SRH were less likely to agree to take part in an interview. Exclusion of people with significant aphasia and those with cognitive deficit (due to being unable to provide informed consent), means that our findings are therefore largely limited to those who have made better recoveries following the stroke. Further research investigating self-rated health perceptions among stroke survivors with severe aphasia and whether these are influenced by factors other than those identified in this study, would be important in informing post-stroke care of survivors with aphasia. Interviews in this study were also conducted only with individuals with a first diagnosed stroke, which may not always be applicable to those who have survived multiple strokes.

We aimed to recruit and interview a purposive sample of stroke survivors with a range of socio-demographic characteristics. However due to the catchment area of the study, there was some homogeneity, for example, a higher number of older individuals of white ethnic backgrounds and higher social class took part. Whilst the views of carers who were present at the interview were not used as data in this analysis, carers’ presence may have influenced some participants’ willingness to express fully their views. On the other hand, many participants appeared more comfortable to speak to the interviewer in the presence of their carer. Our study also provides only cross sectional information based on individual interviews, so we were unable to assess how influences on SRH change over time. Furthermore, we carried out interviews with stroke survivors at between 4 to 6 months following their stroke to minimise variations in different stages of recovery between participants and to ensure early recovery had taken place by the time of interview. We acknowledge that there may have been subtle changes in self-rated health assessment during the period between SRH measurement and interview. It is also uncertain whether our observations focusing on subjective health perceptions in stroke survivors would remain true for participants in the longer-term.

## Conclusions

Patient-centred perceptions of SRH are important in predicting future health outcomes. Subjective appraisals of health after stroke are multidimensional reflecting a combination of physical, psychological and social influences, and past and future perceptions of health, rather than related only to current disability levels among stroke survivors. Such contextual factors may help to explain an apparent disability paradox in the accounts of stroke survivors’ perceptions of their own health. As well as focusing on physical functioning, attention to contextual factors influencing long-term adjustment including psychological outlook, sense of control and availability of social support are important to long-term recovery. We propose that targeted interventions to enhance subjective health after stroke, including optimising psychological resilience, coping and social support are integral components of effective programmes of post-stroke rehabilitation.

## Abbreviations

BI: Barthel Index
HADS: Hospital Anxiety Depression Scale
IMD: Index of Multiple Deprivation
MMSE: Mini mental state examination
NHS: National Health Service
REC: Regional Ethics Committee
SRH: Self-rated health
